# Dietary patterns of persons with chronic conditions within a multi-ethnic population: results from the nationwide Knowledge, Attitudes and Practices survey on diabetes in Singapore

**DOI:** 10.1186/s13690-022-00817-2

**Published:** 2022-02-21

**Authors:** Yeow Wee Brian Tan, Jue Hua Lau, PV AshaRani, Kumarasan Roystonn, Fiona Devi, Ying Ying Lee, Clare Whitton, Peizhi Wang, Saleha Shafie, Sherilyn Chang, Anitha Jeyagurunathan, Boon Yiang Chua, Edimansyah Abdin, Chee Fang Sum, Eng Sing Lee, Mythily Subramaniam

**Affiliations:** 1 Research Division, Institute of Mental Health, 10 Buangkok View, Buangkok Green Medical Park, 539747 Singapore, Singapore; 2 School of Public Health, Faculty of Health Sciences, Curtin University, Kent Street, Western Australia 6102 Bentley, Australia; 3 Admiralty Medical Centre, Khoo Teck Puat Hospital, 676 Woodlands Drive 71, Singapore, Singapore; 4 National Healthcare Group Polyclinics, Fusionopolis Link. Nexus@One-North, Singapore, Singapore

**Keywords:** Chronic conditions, Dietary patterns, DASH, Singapore

## Abstract

**Background:**

Chronic conditions are a leading cause of death and disability worldwide and respective data on dietary patterns remain scant. The present study aimed to investigate dietary patterns and identify sociodemographic factors associated with Dietary Approaches to Stop Hypertension (DASH) scores within a multi-ethnic population with various chronic conditions.

**Methods:**

The present study utilised data from the 2019-2020 Knowledge, Attitudes, and Practices study on diabetes in Singapore – a nationwide survey conducted to track the knowledge, attitudes, and practices pertaining to diabetes. The study analysed data collected from a sample of 2,895 Singapore residents, with information from the sociodemographic section, DASH diet screener, and the modified version of the World Mental Health Composite International Diagnostic Interview (CIDI) version 3.0 checklist of chronic physical conditions.

**Results:**

Respondents with no chronic condition had a mean DASH score of 18.5 (±4.6), those with one chronic condition had a mean DASH score of 19.2 (±4.8), and those with two or more chronic conditions had a mean DASH score of 19.8 (±5.2). Overall, the older age groups [35– 49 years (B = 1.78, 95% CI: 1.23 – 2.33, *p* <0.001), 50–64 years (B = 2.86, 95% CI: 22.24 – 3.47, *p* <0.001) and 65 years and above (B = 3.45, 95% CI: 2.73 – 4.17, *p* <0.001)], Indians (B = 2.54, 95% CI: 2.09 – 2.98, *p* <0.001) reported better diet quality, while males (B = -1.50, 95% CI: -1.87 – -1.14, *p* <0.001) reported poorer diet quality versus females.

**Conclusion:**

Overall, respondents with two or more chronic conditions reported better quality of diet while the sociodemographic factors of age, gender and ethnicity demonstrated a consistent pattern in correlating with diet quality, consistent with the extant literature. Results provide further insights for policymakers to refine ongoing efforts in relation to healthy dietary practices for Singapore.

## Background

According to the US Department of Health and Human Services, chronic conditions are defined as “conditions lasting a year or more that require ongoing medical attention and/or limit activities of daily living” [1]. Chronic conditions are an ongoing cause of substantial ill health, disability, and premature death, making them an important global, national, and individual health concern [2]. Based on the latest statistics from the World Health Organization, it is estimated that the contribution of major chronic conditions toward death and global burden of disease is approximately 71% as of 2021 [3].

Globally, one in three adults live with multiple chronic conditions (MCC) [4], a figure that is expected to rise significantly with studies suggesting that the proportion of patients with four or more diseases is expected to double by 2035 in countries such as the United Kingdom [5]. MCC has long been associated with adverse health outcomes, with individuals experiencing early mortality [6], poorer physical and mental health [7] and reduced quality of life [8]. Additionally, given the complexities of clinical treatment and patient management, MCC is consequently associated with higher resource utilisation and increased medical costs [9, 10]. Specifically, average per capita health care spending increases exponentially with the number of chronic conditions – a figure estimated at  $1,081 for people with no chronic conditions to $5,074 for those with two chronic conditions and $14,768 for people with five or more chronic conditions [11].

The etiology of chronic conditions is complex and multifactorial. Risk factors include age, family history, genetic predisposition, current and lifetime weight and physical activity, smoking, alcohol, and diet [12, 13]. Of these risk factors, dietary choices may arguably be the most amenable to modification and with the greatest public health impact [14]. Of note, Sofi et al. [15] highlighted that individuals reporting a greater degree of adherence to healthier dietary intake such as the Mediterranean diet showed a significant protection against the development of chronic conditions; with approximately 6-13% reduction in deaths and/or incidence of neurodegenerative disease, cardiovascular disease and cancer. Evidently, such a dietary pattern is recognised as a major contributing factor towards the prevention, development and treatment of chronic conditions [15]. Correspondingly, a growing and evolving body of scientific inquiry on dietary intake with health and chronic conditions has led to recommendations emphasizing a variety of plant-based foods (e.g., vegetables, fruits, legumes, whole grains, nuts, and seeds) and de-emphasising processed food consumption with added sugar or a diet that is rich in meat content [14, 16].

Studies in Asian populations have identified various dietary patterns labelled “traditional,” “meat,” “Western,” and “prudent”. Dietary patterns observed in China characterised by a high intake of meat and dairy products have been associated with obesity [17, 18]. In Thailand, a more traditional carbohydrate-rich pattern was associated with metabolic syndrome [19]. In other populations such as Japan and Korea, a more traditional dietary pattern was inversely associated with risk factors such as high blood pressure [20, 21]. In Pakistan where 33% of the adult population suffers from hypertension, the high intake of fish, prawns and yoghurt was found to be inversely associated with hypertension [22]. Within the Singapore Chinese population, a “fruit-vegetable-soy” pattern was inversely associated, and a “meat-dim-sum” pattern was directly associated with cardiovascular disease mortality [23]. Evidently, the association between various dietary patterns and chronic conditions has been established within singular ethnic populations across the considerable corpus of research in this area. Yet, much less is known regarding the association between healthy dietary patterns and chronic conditions across a multi-ethnic population.

Singapore is a multi-ethnic city-state situated in Southeast Asia with a population of approximately 5.6 million of which 4.1 million are Singapore residents (Singapore citizens or permanent residents) [24]. The population is largely comprised of inhabitants from three major Asian ethnic groups: Chinese (76.0%), Malay (15.0%) and Indian (7.5%) [25]. Based on the results from the Singapore Mental Health Study, it was found that a total of 25.4% individuals reported having one chronic condition, and 16.3% had MCC [26]. Given the relatively substantial figures of individuals living with chronic conditions, it underscores the need to address the paucity of dietary data in this population. Additionally, a study in this setting provides a unique opportunity to elucidate the dietary patterns of a multi-ethnic population, the results of which can be extrapolated to other countries with a similar ethnic composition.

Given the diverse ethnic composition in Singapore, it follows that a culturally relevant diet screener is required for use in this multi-ethnic population. Whitton et al. [27] developed a reliable and validated short diet screener designed to assess the intake of selected food groups that is representative of the overall dietary patterns across a multi-ethnic Asian population. Specifically, the short 37-item diet screener assesses the intake of selected food groups representative of a multi-ethnic Asian population via *a priori* dietary quality indices such as DASH. In the extant literature, DASH has been demonstrated to be the most sensitive diet score to examine associations between diet and various health-related outcomes [28]. Adopting DASH dietary patterns has several benefits including but not limited to lowered mortality from cardiovascular diseases and diabetes [28, 29], lowered blood pressure [30], decreased body weight and waist circumference in obesity related weight management [31].

Taken together, the aims of the present study were to (1) characterise and examine the dietary patterns of a multi-ethnic population between persons with no chronic condition, one chronic condition and MCC through scoring their dietary intake according to the DASH score, and (2) identify socio-demographic correlates of DASH scores amongst persons with no chronic condition, one chronic condition and MCC.

## Methods

### Participants and procedures

The data for this research comes from a population based, cross-sectional study lasting from February 2019 to September 2020 aimed at evaluating the Knowledge, Practice and Attitudes towards Diabetes Mellitus (DM) amongst residents of Singapore aged 18 years and above. A more to detailed methodology of the study can be found in an earlier paper [32]. The sample was randomly selected via a disproportionate stratified sampling design according to ethnicity (Chinese, Malay, Indian, Others) and age groups (18-34, 35-49, 50-64, 65 and above) from a national population registry database of all citizens and permanent residents within Singapore. The study oversampled certain minority populations, such as Malay and Indian ethnicity, as well as those above 65 years of age, in order to ensure sufficient sample size and to improve the reliability of the parameter estimates for these subgroups.

Citizens and permanent residents who were randomly selected were sent notification letters followed by home visits by a trained interviewer from a survey research company to obtain their informed consent to participate in the study. Face-to-face interviews with those who were agreeable to participate were conducted in their preferred language (English, Mandarin, Malay, or Tamil). Responses were captured using computer assisted personal interviewing. Individuals who were unable to be contacted due to incomplete or incorrect addresses, were living outside of the country, or were incapable of attending the interview due to severe physical or mental conditions, language barriers, or were institutionalized or hospitalized at the time of the survey were excluded from the study. Written informed consent was obtained from all respondents prior to the survey, and for those aged 18 to 20 years, parental consent was sought as the official age of majority in Singapore is 21 years and above.

### Measures

#### Socio-demographic information and body mass index

Socio-demographic data on age (18-34, 35-49, 50-64 and 65 and above), gender (Female, Male), ethnicity (Chinese, Malay, Indian and Others), education (Primary and below, Secondary, Pre-U/Junior College, Vocational Institute/ITE, Diploma, Degree, professional certifications and above), marital status (Single, Married/Cohabiting, Divorced/Separated/Widowed), employment (Employed, Economically inactive and Unemployed), and monthly personal income (Below $2,000 and no income, $2,000-$3,999, $4,000-$5,999, $6000-$9,999 and $10,000 and above) was collected. Further, Body Mass Index (BMI) scores were categorised into four groups based on World Health Organization guidelines: ‘underweight (< 18.5 kg/m^2^), ‘normal range’ (≥ 18.5 kg/m^2^ and < 25 kg/m^2^), ‘overweight’ (≥ 25 kg/m^2^ and < 30 kg/m^2^), and ‘obese’ (>30 kg/m^2^) [33].

### Diet screener

The diet screener comprises a list of 30 food/beverage items, that respondents’ rate on a 10-point scale ranging from ‘never/rarely’ to ‘6 or more times per day’, the frequency at which they consumed a particular food/beverage within the last one year [27]. Accordingly, scores obtained from the list of 30 food/beverage items are categorised into each of the seven intake components: fruit, vegetables, nuts/legumes, whole grains, red and processed meat, low fat dairy, and sweetened beverages. Based upon the scores within each of the seven components, participants received a score between 1 and 5 corresponding to the quintile of the intake they fall in, with reverse scoring utilized for meat and sweetened beverages. Followingly. these seven quintile scores are summed to form the overall DASH score. Standard serving sizes were indicated for each food/beverage item to facilitate this process. Intake frequencies were standardised to a number of servings per day for each food/beverage item. Fung et al. [34] provides a detailed description on the calculation of DASH scores. The diet screener was interviewer-administered.

### Chronic physical conditions

A modified version of the World Mental Health Composite International Diagnostic Interview (CIDI) version 3.0 [35] checklist of chronic conditions was used, and the respondents were asked to report any of the conditions listed in the checklist. The question was read as, “I am going to read to you a list of health problems some people have. Has a doctor ever told you that you have any of the following chronic medical conditions?” This was followed by a list of 18 chronic physical conditions (such as asthma, high blood sugar, hypertension, arthritis, cancer, neurological condition, Parkinson’s disease, stroke, congestive heart failure, heart disease, back problems, stomach ulcer, chronic inflamed bowel, thyroid disease, kidney failure, migraine headaches, chronic lung disease, and hyperlipidaemia) which were considered to be prevalent among Singapore’s population. If the participant gave a positive response for any of the conditions listed, they were then asked, “How old were you when you were diagnosed with the medical condition?” and, “Did you receive any treatment for it at any time during the past 12 months?”.

### Statistical analysis

Analyses in the present study were conducted with Stata version 15. In order to ensure representativeness of the data to the general population, survey weights were used to account for complex survey design. Means and standard deviations are provided for continuous variables, while frequencies and percentages are presented for categorical variables. In order to examine the variables associated with the total DASH score, four linear regressions were conducted (within the total sample, and the three subgroups of no chronic condition, one chronic condition and multimorbidity) with the following predictor variables: age, gender, ethnicity, education, marital status, employment status, personal income, BMI and chronic conditions. Statistical significance was set at the conventional alpha level of *p* < 0.05, using two-tailed tests.

## Results

### Socio-demographics distribution and prevalence of physical comorbidities of the sample

Table 1 summarises the socio-demographic characteristics for the sample of 2,895 respondents. Chinese respondents made up 75.8% of the sample distribution, Malays 12.7%, Indians 8.6%, and Others 2.9%. 51.6% of the respondents were female, and BMI scores indicated that 53.4% of the respondents were in the normal range based on WHO BMI classification. 46.2% had no chronic physical condition, 26.3% had one chronic physical condition, and 27.2% had two or more chronic physical conditions. The most prevalent chronic condition was hyperlipidemia (22.2%, n=640), followed by hypertension (20.6%, n=668), high blood sugar (10.5%, n=465), back problems (10.2%, n=252), and asthma (9.9%, n=324). The results describing the prevalence of the different types of chronic physical conditions are displayed in Table 2.

**Table 1:** Socio-demographic distribution of the sample, Singapore, 2019-2020, Knowledge, Attitudes, and Practices study on diabetes

Socio-demographic characteristics	Total sample (n = 2895)		No chronic physical condition (n = 1243) (Weighted = 46.21%)		One chronic physical condition (n = 760) (Weighted = 26.32%)		Two or more chronic physical conditions (n = 884) (Weighted = 27.21%)	
	n	Weighted %	n	Weighted %	n	Weighted %	n	Weighted %
Age groups								
18-34	823	29.89	537	42.63	207	29.06	78	9.34
35-49	719	28.22	369	31.45	215	30.50	135	20.86
50-64	774	26.75	239	18.27	203	27.48	328	40.01
65 and above	579	15.13	98	7.65	135	12.96	343	29.79
Gender								
Female	1,474	51.55	605	51.64	416	54.96	448	47.63
Male	1,421	48.45	638	48.36	344	45.04	436	52.37
Ethnicity								
Chinese	796	75.81	360	75.15	206	75.36	227	77.28
Malay	974	12.73	410	12.97	251	12.47	310	12.60
Indian	918	8.59	375	8.64	243	8.86	298	8.31
Others	207	2.86	98	3.24	60	3.31	49	1.81
Education								
Primary and below	637	20.36	160	12.30	163	18.91	309	35.00
Secondary	684	20.28	272	20.14	162	18.41	248	22.17
Pre-U^a^/Junior College	126	4.78	60	5.51	34	4.56	32	3.80
Vocational Institute/ITE^b^	267	6.62	132	7.17	76	7.91	58	4.46
Diploma	479	18.46	267	21.85	124	19.10	88	12.31
Degree, professional certifications and above	702	29.50	352	33.04	201	31.13	149	22.26
Marital Status								
Single	731	29.20	443	39.10	182	27.50	105	14.30
Married/Cohabiting	1,860	61.69	721	54.49	501	66.10	633	69.97
Divorced/Separated/Widowed	303	9.10	78	6.40	77	6.40	146	15.72
Refused	1	0.01	1	0.01	0	0.00	0	0.00
Employment								
Employed	1,933	70.49	922	77.16	525	72.22	482	57.56
Economically inactive	829	25.37	263	19.05	204	24.39	358	36.94
Unemployed	133	4.14	58	3.79	31	3.39	44	5.50
Personal Income (Monthly)^c^								
Below 2,000/No income	1,455	45.31	545	40.26	366	44.62	537	54.27
2,000-3,999	698	23.94	334	26.59	190	23.19	173	20.09
4,000-5,999	318	12.77	162	13.74	90	14.21	66	9.90
6,000-9,999	183	7.82	92	8.89	47	6.79	44	7.09
10,000 and above	117	5.68	45	5.25	36	6.88	36	5.31
Don’t know/Refused	124	4.48	65	5.28	31	4.31	28	3.34
BMI^d^								
Normal range ≥ 18.5 & <25	1263	53.39	617	55.80	346	59.31	298	43.78
Underweight < 18.5	151	6.95	90	10.04	40	6.16	20	2.17
Overweight ≥ 25 & <30.0	858	26.57	338	23.80	222	25.22	296	32.55
Obese ≥ 30.0	420	8.97	150	7.63	100	6.58	168	13.62
Refused	203	4.13	48	2.72	52	2.73	102	7.89
Classifications								
No chronic physical condition	1,243	46.21	-	-	-	-	-	-
One chronic physical condition	760	26.28	-	-	-	-	-	-
Two or more chronic physical conditions	884	27.18	-	-	-	-	-	-

^a^*Pre-U* Pre-University

^b^*ITE* Institute of Technical Education

^c^Denoted in Singapore Dollars (SGD). USD1 ≈ SGD1.4

^d^*BMI* Body Mass Index

**Table 2:** Prevalence of chronic physical condition in the sample, Singapore, 2019-2020, Knowledge, Attitudes, and Practices study on diabetes

Chronic physical conditions	n	Weighted %
Overall prevalence		
Hyperlipidaemia or high cholesterol	640	22.22
Hypertension or high blood pressure	668	20.55
High blood sugar or diabetes	465	10.47
Back problems including disk or spine	252	10.20
Asthma	324	9.93
Migraine headaches	278	7.87
Arthritis or rheumatism	173	5.90
Heart disease (including a heart attack, coronary heart disease, angina, or other heart disease)	150	3.83
Thyroid disease	107	3.32
Cancer	56	1.59
Stomach ulcer	45	1.50
Stroke or major paralysis (inability to use arms or legs)	32	0.90
Kidney failure	32	0.77
Chronic lung diseases such as chronic bronchitis or emphysema (excluding Asthma)	19	0.66
A neurological condition, such as epilepsy or convulsions	12	0.38
Congestive heart failure	24	0.36
Chronic inflamed bowel, enteritis, or colitis	6	0.14
Parkinson’s disease	4	0.09

### DASH components score distribution and comparisons between chronic physical condition groups

The means and standard deviations of each of the seven DASH components (fruit, vegetables, nuts/legumes, low fat dairy, whole grains, red and processed meat, and sweetened beverages) and the overall DASH scores of the total sample, those with no chronic physical condition, one chronic physical condition and two or more chronic physical conditions are displayed in Table 3.

**Table 3:** Dietary Approaches to Stop Hypertension components and overall scores of no chronic physical condition, one physical chronic condition and two or more chronic physical conditions, Singapore, 2019-2020, Knowledge, Attitudes, and Practices study on diabetes

		Total sample (*n* = 2895)	No chronic physical condition (*n* = 1243) (Weighted = 46.21%)	One chronic physical condition (*n* = 760) (Weighted = 26.32%)	Two or more chronic physical conditions (*n* = 884) (Weighted = 27.21%)
DASH^a^ components (servings/d)		Mean (S.D.)			
	Fruits	0.96	0.89	1.02	1.03
		(0.91)	(0.84)	(0.96)	(0.95)
	Vegetables	1.57	1.52	1.62	1.60
		(1.14)	(1.12)	(1.14)	(1.19)
	Nuts/Legumes	0.67	0.61	0.77	0.65
		(0.82)	(0.75)	(0.91)	(0.85)
	Low fat dairy	0.29	0.29	0.29	0.31
		(0.47)	(0.46)	(0.45)	(0.49)
	Whole grains	1.63	1.51	1.73	1.75
		(1.87)	(1.72)	(2.10)	(1.88)
	Red and processed meat	0.57	0.59	0.58	0.51
		(0.66)	(0.67)	(0.68)	(0.63)
	Sweetened beverages	0.34	0.36	0.37	0.27
		(0.52)	(0.52)	(0.56)	(0.47)
DASH score^b^		19.05	18.54	19.19	19.75
		(4.84)	(4.57)	(4.83)	(5.17)

^a^Dietary Approaches to Stop Hypertension

^b^DASH scores are calculated based on quintile score from each of the seven components

### Socio-demographic correlates of DASH score

Table 4 shows the socio-demographic correlates associated with DASH scores within the full sample and across the three chronic physical condition groups. Within the full sample, the older age group [35– 49 years (B = 1.78, 95% CI: 1.23 – 2.33, *p* <0.001), 50–64 years (B = 2.86, 95% CI: 2.24 – 3.47, *p* <0.001) and 65 years and above (B = 3.45, 95% CI: 2.73 – 4.17, *p* <0.001)] had significantly higher DASH scores as compared to those aged 18-34. Males reported significantly lower DASH scores (B = -1.50, 95% CI: -1.87 – -1.14, *p* <0.001) than females. Indians (B = 2.54, 95% CI: 2.09 – 2.98, *p* <0.001) had significantly higher DASH scores than Chinese. Respondents who were less educated [primary and below (B = -1.99, 95% CI: -2.69 – -1.29, *p* <0.001), secondary (B = -1.72, 95% CI: -2.31 – -1.12, *p* <0.001), vocational institute/ITE (B = -1.42, 95% CI: -2.15 – -0.69, *p* <0.001), and diploma (B = -0.84, 95% CI: -1.40 – -0.27, *p* =0.004)] had significantly lower DASH scores than those with degree, and above qualifications.

**Table 4:** Results of the linear regression analyses examining the correlates of the number of chronic physical conditions and Dietary Approaches to Stop Hypertension scores, Singapore, 2019-2020, Knowledge, Attitudes, and Practices study on diabetes

	DASH score					
	Total sample (*n* = 2571)			No chronic physical condition (*n* = 1133)		
	B	95% CI	*p*	B	95% CI	*p*
Age groups						
18-34	ref			ref		
35-49	1.78	1.23 – 2.33	**<0.001**	1.77	1.02 – 2.52	**<0.001**
50-64	2.86	2.24 – 3.47	**<0.001**	2.38	1.47 – 3.28	**<0.001**
65 and above	3.45	2.73 – 4.17	**<0.001**	3.41	2.20 – 4.63	**<0.001**
Gender						
Female	ref			ref		
Male	-1.50	-1.87 – -1.14	**<0.001**	-1.89	-2.42 – -1.35	**<0.001**
Ethnicity						
Chinese	ref			ref		
Malay	0.26	-0.20 – 0.72	0.27	0.45	-0.23 – 1.13	0.19
Indian	2.54	2.09 – 2.98	**<0.001**	2.65	1.99 – 3.31	**<0.001**
Others	0.67	-0.05 – 1.39	0.07	0.96	-0.07 – 1.98	0.07
Education						
Degree, professional certifications and above	ref			ref		
Primary and below	-1.99	-2.69 – -1.29	**<0.001**	-2.06	-3.23 – -0.88	**0.001**
Secondary	-1.72	-2.31 – -1.12	**<0.001**	-1.42	-2.28 – -0.57	**0.001**
Pre-U/Junior College	-0.39	-1.28 – 0.51	0.40	0.82	-0.48 – 2.11	0.22
Vocational Institute/ITE	-1.42	-2.15 – -0.69	**<0.001**	-1.75	-2.77 – 0.73	**0.001**
Diploma	-0.84	-1.40 – -0.27	**0.004**	-0.64	-1.42 – 0.13	0.11
Marital Status						
Married/Cohabiting	ref			ref		
Single	-0.28	-0.79 – 0.24	0.30	-0.17	-0.89 – 0.55	0.64
Divorced/Separated/Widowed	-0.55	-1.16 – -0.07	0.08	-0.29	-1.41– 0.83	0.61
Employment						
Employed	ref			ref		
Economically inactive	0.45	-0.03 – 0.93	0.07	0.60	-0.16 – 1.37	0.12
Unemployed	0.39	-0.44 – 1.23	0.35	0.35	-0.89 – 1.59	0.58
Personal Income (Monthly)						
Below 2,000/No income	ref			ref		
2,000-3,999	-0.30	-0.79 – 0.19	0.23	-0.10	-0.82 – 0.62	0.78
4,000-5,999	-0.27	-0.92 – 0.38	0.42	-0.17	-1.11 – 0.77	0.73
6,000-9,999	-0.03	-0.85 – 0.79	0.95	0.43	-0.76 – 1.61	0.48
10,000 and above	0.13	-0.86 – 1.12	0.80	0.15	-1.41 – 1.71	0.85
BMI						
Normal range ≥ 18.5 & <25	ref			ref		
Underweight < 18.5	-0.14	-0.90 – 0.62	0.81	-0.41	-1.38 – 0.56	0.41
Overweight ≥ 25 & <30.0	0.22	-0.18 – 0.63	0.27	0.65	0.04 – 1.25	0.05
Obese ≥ 30.0	-0.17	-0.68 – 0.34	0.52	-0.16	-0.97 – 0.65	0.70
Classifications						
No chronic physical condition	ref			-	-	-
One chronic physical condition	0.04	-0.38 – 0.46	0.84	-	-	-
Two or more chronic physical conditions	0.14	-0.31 – 0.59	0.54	-	-	-
	DASH score					
	One chronic physical condition (*n* = 681)			Two or more chronic physical conditions (*n* = 757)		
	B	95% CI	*p*	B	95% CI	*p*
Age groups						
18-34	ref			ref		
35-49	1.54	0.45 – 2.63	**0.01**	2.90	1.52 – 4.27	**<0.001**
50-64	2.87	1.67 – 4.07	**<0.001**	4.10	2.78 – 5.41	**<0.001**
65 and above	4.19	2.76 – 5.63	**<0.001**	3.87	2.45 – 5.29	**<0.001**
Gender						
Female	ref			ref		
Male	-0.97	-1.71 – -0.22	**0.01**	-1.48	-2.19 – -0.77	**<0.001**
Ethnicity						
Chinese	ref			ref		
Malay	-0.47	-1.40 – 0.46	0.32	0.61	-0.27 – 1.49	0.18
Indian	2.19	1.30 – 3.09	**<0.001**	2.61	1.78 – 3.44	**<0.001**
Others	-0.35	-1.75 – 1.04	0.62	1.49	0.01 – 2.97	**0.04**
Education						
Degree, professional certifications and above	ref			ref		
Primary and below	-3.04	-4.39 – -1.69	**<0.001**	-1.04	-2.33 – 0.26	0.12
Secondary	-2.35	-3.54 – -1.16	<**0.001**	-1.19	-2.41 – 0.03	0.06
Pre-U/Junior College	-1.09	-2.85 – 0.68	0.23	-1.44	-3.25 – 0.38	0.12
Vocational Institute/ITE	-1.22	-2.61 – 0.17	0.08	-0.76	-2.38 – 0.86	0.36
Diploma	-1.51	-2.61 – -0.40	**0.01**	-0.22	-1.56 – 1.11	0.74
Marital Status						
Married/Cohabiting	ref			ref		
Single	-0.53	-1.60 – 0.54	0.33	-0.18	-1.28 – 0.91	0.74
Divorced/Separated/Widowed	-0.66	-1.94 – 0.62	0.31	-0.68	-1.60 – 0.25	0.15
Employment						
Employed	ref			ref		
Economically inactive	0.26	-0.69 – 1.22	0.59	0.34	-0.55 – 1.23	0.45
Unemployed	0.97	-0.81 – 2.74	0.29	-0.07	-1.56 – 1.43	0.93
Personal Income (Monthly)						
Below 2,000/No income	ref			ref		
2,000-3,999	-0.32	-1.27 – 0.63	0.51	-0.54	-1.49 – 0.41	0.26
4,000-5,999	0.41	-0.85 – 1.67	0.52	-1.41	-2.79 – -0.04	0.04
6,000-9,999	-0.12	-1.74 – 1.50	0.89	-0.59	-2.26 – 1.09	0.49
10,000 and above	-0.66	-2.50 – 1.17	0.48	1.01	-0.82 – 2.85	0.28
BMI						
Normal range ≥ 18.5 & <25	ref			ref		
Underweight < 18.5	-0.12	-1.65 – 1.40	0.87	0.68	-1.37 – 2.73	0.52
Overweight ≥ 25 & <30.0	0.21	-0.57 – 1.00	0.60	-0.44	-1.19 – 0.31	0.25
Obese ≥ 30.0	0.85	-0.20 – 1.89	0.11	-1.06	-1.94 – 0.18	0.20
Classifications						
No chronic physical condition	-	-	-	-	-	-
One chronic physical condition	-	-	-	-	-	-
Two or more chronic physical conditions	-	-	-	-	-	-

B – represents unstandardized coefficient; 95% CI: 95% confidence interval of β

Bold print denotes statistically significant B value

Numbers included within analyses may not tally with sample size due to missing data on variables

Across all three groups, the older age groups reported [no chronic physical condition group: 35– 49 years (B = 1.77, 95% CI: 1.02 – 2.52, *p* <0.001), 50–64 years (B = 2.38, 95% CI: 1.47 – 3.28, *p* <0.001) and 65 years and above (B = 3.41, 95% CI: 2.20 – 4.63, *p* <0.001), one chronic physical condition group: 35– 49 years (B = 1.54, 95% CI: 0.45 – 2.63, *p* =0.01), 50–64 years (B = 2.87, 95% CI: 1.67 – 4.07, *p* <0.001) and 65 years and above (B = 4.19, 95% CI: 2.76 – 5.63, *p* <0.001) and two or more chronic physical condition group: 35– 49 years (B = 2.90, 95% CI: 1.52 – 4.27, *p* =0.001), 50–64 years (B = 4.10, 95% CI: 2.78 – 5.41, *p* <0.001) and 65 years and above (B = 3.87, 95% CI: 2.45 – 5.29, *p* <0.001)] significantly higher DASH scores as compared to those aged 18-34.

Males reported [no chronic physical condition group: (B = -1.89, 95% CI: -2.42 – -1.35, *p* <0.001), one chronic physical condition group: (B = -0.97, 95% CI: -1.71 – -0.22, *p* =0.01) and two or more chronic physical condition (B = -1.48, 95% CI: -2.19 – -0.77, *p* <0.001)] significantly lower DASH scores than females.

Respondents of Indian ethnicity reported [no chronic physical condition group: (B = 2.65, 95% CI: 1.99 – 3.31, *p* <0.001), one chronic physical condition group: (B = 2.19, 95% CI: 1.30 – 3.09, *p* <0.001) and two or more chronic physical condition group: (B = 2.61, 95% CI: 1.78 – 3.44, *p* <0.001)] significantly higher DASH scores than Chinese.

In the no chronic physical condition group, respondents who were less educated [primary and below (B = -2.06, 95% CI: -3.23 – -0.88, *p* =0.001), secondary (B = -1.42, 95% CI: -2.28 – -0.57, *p* =0.001), vocational institute/ITE (B = -1.75, 95% CI: -2.77 – 0.73, *p* =0.001)] had significantly lower DASH scores than those with degree, professional certification and above. Similarly, respondents with lower education in the one chronic physical condition group [primary and below (B = -3.04, 95% CI: -4.39 – -1.69, *p* <0.001), secondary (B = -2.35, 95% CI: -3.54 – -1.16, *p* <0.001), diploma (B = -1.51, 95% CI: -2.61 – -0.40, *p* =0.01)] had significantly lower DASH scores in contrast to their counterparts with degree, professional certification and above. In the two or more chronic physical condition group, respondents of “Others” ethnicities (B = 1.49, 95% CI: 0.01 – 2.97, *p* =0.04) demonstrated significantly higher DASH scores than Chinese ethnicity.

## Discussion

The current study found that 42.9% of the population had no chronic physical condition, 26.3% had one chronic physical condition, and 30.5% had MCC. Overall, people with MCC had demonstrated better dietary practices in terms of number of servings taken per day for the DASH components. In the context of Singapore, this could be attributed to various initiatives and support received from primary care providers. Firstly, the focus upon nudging and facilitating healthier dietary choices is seen across the multitude of nationwide health promotion campaigns. One such example relates to the “Healthier Dining Programme” launched in 2014 which provides incentives to restaurants offering 500-calorie meals. Essentially, consumers are “nudged” towards choosing such healthier meal options that are identified with a “Healthier Choice Symbol” on menus in these restaurants (36). More importantly, better dietary practices amongst persons with MCC can also be attributed to the role of primary care providers as outlined in several guidelines and regulations. Of relevance, the “Chronic Disease Management Programme” introduced earlier in 2006 represents one avenue aiding in the management of chronic physical conditions in Singapore [37]. Briefly, the programme involves structured disease management aimed at reducing out-of-pocket payments for outpatient treatments required in the management of an individual’s chronic diseases [38]. In terms of dietary habits, healthcare professionals (e.g., physicians and dieticians) provide individuals with practical dietary guidelines aimed at making adjustments to current dietary choices [39]. Taken together, the combination of both healthier dietary practices campaigns and prevention efforts in the clinical setting provide plausible explanations to the present finding of better dietary patterns observed in persons with MCC.

Findings from the present study lends further support to several well-established risk factors associated with diet quality in the extant literature. Within the study population, females and those of older age reported better diet quality based on their respective DASH scores. In a study determining the demographic profile of fast-food consumers amongst a Singapore population, Whitton et al. [40] reported that older adults consumed lesser fast food in comparison to their younger counterparts. As highlighted by Allman-Farinelli et al. [41], this is consistent with the notion that younger individuals demonstrate certain dietary habits that reduce overall diet quality. Food and beverages with high saturated fat, sugar and sodium contents such as those purchased at quick service restaurants feature prominently in the younger population’s diet across many countries such as USA, UK and Australia [41].

Gender differences in terms of dietary patterns was also demonstrated to be consistent with prior studies; where women tended to report better diet quality in comparison to men. For example, in Montreal, women’s diets were closer to recommendations for vegetables, fruits, and sodium intake as compared to men [42]. As demonstrated in prior literature, women generally reported being more invested in relation to food-related matters and having better knowledge in terms of food and nutrition [43, 44]. Additionally, women reported consuming higher intakes of fruits and vegetables, dietary fibre, and lower intakes of fat and salt [43, 45]. Taken together, it follows that greater importance attributed by women to their diet correspondingly translates into better dietary practices.

Across the sample population, ethnicity was also identified to be significantly associated with diet quality. Among the major ethnic groups in Singapore, Indians reported having healthier diet based on respective DASH scores. This is consistent with the healthy dietary pattern as outlined in the National Nutrition Survey conducted in 2010 [46], with Indians consuming the most bread and breakfast cereals, vegetable dishes, fruit, milk and dairy products and fewer eggs, poultry and meat dishes.

Diet and nutrition represent important factors in both promotion and maintenance of good health throughout the entire life course. We identified several important factors associated with diet quality among persons with no chronic conditions and one chronic condition. Within these subgroups, education level was significantly associated with diet quality. Specifically, those with less than a degree reported having poorer diet quality. Similar findings regarding educational level have been reported elsewhere; adults with a college diploma in USA demonstrated having a better overall diet quality as compared to all other education levels [47]. Therein, it has been posited that education might be associated not only with increased nutritional knowledge, but could also be an indicator of ability to translate such nutritional knowledge into better dietary practices throughout the person’s lifetime [47]. Lastly, it was interesting to observe that BMI was not a factor associated with diet quality in the present study. In current literature, it has been evidenced that individuals with chronic conditions generally reported higher-than-normal BMI (48). Accordingly, multiple studies have also demonstrated that healthier dietary patterns are associated with lower BMI [49, 50]. Nonetheless, it should be noted that the use of self-reported chronic conditions may plausibly have resulted in reporting bias – underestimating the true prevalence of chronic conditions and therein, an underestimation of the strength of the association between BMI and diet quality.

The present study was a nationwide survey conducted in four different languages (English, Chinese, Malay and Tamil) to address potential language barriers for participation in a multi-ethnic population. Additionally, the use of a large sample size, randomised design and survey weighted analysis improves the overall reliability of the present results. Nonetheless, several limitations of the present study warrant comment and conclusions drawn should be considered in light of these limitations. Firstly, given that the present study adopts a cross-sectional design, we are not able to establish any causal relationship between dietary pattern and chronic conditions. Secondly, the diet screener utilised is fundamentally based on the past-year self-reported diet recall of the respondent, with no correlation to any blood or urinary parameters. In that regard, we are not able to rule out the likelihood of recall bias in this self-reported format. Additionally, while it has been demonstrated that intakes such as whole grains and fruits can be adequately assessed in the present diet screener, more comprehensive and detailed instruments such as comprehensive food frequency questionnaires are still recommended for the assessment of other dietary components such as nutrients.

## Conclusion

Chronic conditions remain the leading cause of death and disability worldwide. Despite this, it should be noted that chronic conditions are largely preventable, and dietary patterns play a key role in such prevention activities. Given its significance, it reasons that establishing population norms in relation to dietary habits represent an important aspect of research in this area. Given the limited research conducted regarding dietary patterns of a multi-ethnic population, the present study furthers the understanding on the dietary patterns of a multi-ethnic population like Singapore. Notably, while the dietary patterns of individuals with chronic conditions are positive, the present study highlights areas of improvement for better dietary practices amongst individuals without chronic conditions. Given that individuals who are younger and healthy at present tended to have lower DASH scores, it is critical that efforts are directed towards monitoring less-than-ideal dietary practices given its significance towards the development of chronic conditions. As noted earlier, ongoing efforts such as the “Healthier Dining Programme” are initiatives aimed at providing healthier meals for individuals. Results on the dietary intakes as categorised by food groups such as fruits and vegetables provide up-to-date information which policymakers can utilise to modify ongoing initiatives and campaigns if necessary. In the global context, studies and reviews have generally reached the conclusion that there are some similarities in terms of dietary recommendations albeit minor differences when a country’s geographical environment and culture are taken into account [51, 52]. Accordingly, information obtained from the present study may provide certain insights to international counterparts pertaining to the effectiveness of diet related policies and initiatives in Singapore. Such information may be useful in aiding policymakers with modifying or forming new polices to better suit their respective population. Taken together, the present study provides both local and international policymakers with up-to-date information on dietary patterns in Singapore to better support the design and evaluation of policies and initiatives.

## Data Availability

The datasets used and/or analysed during the current study are available from the corresponding author on reasonable request.

## Abbreviations

DASH: Dietary Approaches to Stop Hypertension
CIDI: World Mental Health Composite International Diagnostic Interview
MCC: Multiple Chronic Conditions
DM: Diabetes Mellitus
WHO: World Health Organisation
BMI: Body Mass Index
Pre-U: Pre-University
ITE: Institute of Technical Education
SGD: Singapore Dollars
